# Laceration of the Spleen in Pregnancy

**Published:** 1868-11

**Authors:** J. O. Whitney

**Affiliations:** Pawtucket, R. I.


### Miscellaneous.

### Laceration of the Spleen in Pregnancy.

By J. O. Whitney, M.D., Pawtucket, R. I.

The subject of this notice was a Mrs. S., 34 years of age, eight months advanced in her ninth pregnancy, and a patient of Dr. T. Phelps, of North Attleboro', Mass., by whose request I report the case. She had uniformly enjoyed good health, and nothing whatever occurred to indicate the approach of the fatal event, unless a numbness of one of the upper extremities, and a puffness or bloat of the face which had, a week prior, showed danger *somewhere.* For this plethoric state of the vascular system, she mentioned being bled, but medical advice was not taken.

Dec. 28th, 1867, she ate a hearty supper, retiring as usual, but at 11 o'clock, P. M., was taken with the most violent pain in the region of the stomach. Two hours later she was seen by her physician, who gave anodynes with much relief. Two convulsions took place, resembling puerperal fits; and she had remarked, "I am a going to be sick the same as I was before," referring to a premature labor, attended with three convulsions, for which she had been bled with prompt relief. These two fits were, in all respects, like the three she had had in the former illness, only less severe. This is the belief of her husband, who is an observant man, and the suggestion that they were fainting fits, caused by loss of blood, he would not entertain, and he feels certain that the pulse, prior to the first one, was full and strong, though it soon became small and rapid. The first convulsion lasted about thirty minutes, consciousness not fully returning, though she swallowed some medicine, when she went into another, which lasted an hour and a half. Consciousness then returned; she talked, sat up in bed with aid, and got up and passed urine. On being persuaded by her husband, she again lay down, and expired in about an hour, and five hours from the attack. Dr. Phelps considered the convulsions puerperal, the small and rapid pulse deterring him from the use of the lancet, as urged by the family. Inspection of the uterus showed that labor had not begun. The autopsy, made eight hours after death, revealed a lacerated spleen, from which had escaped four or five pints of dark and still fluid blood. The organ was only a little if any enlarged, the torn part of the color and consistence of black currant jelly.

Sir J. Y. Simpson suggests that enlargement and softening of the spleen are quite common in pregnancy; he had seen a case occurring in a series of pregnancies, the organ in the intervals returning to its normal state.

At a meeting of the Obstetrical Society of Edinburgh, in June, 1866, six cases of rupture of the spleen were reported, some taking place prior, some during, and some subsequent to labor—all fatal, of course. From the fact that one patient invariably recovered from this state of "physiological" enlargement of the spleen, we may infer that it is not in a state of disorganization, but only in a condition predisposing to the accident of laceration, and when combined with a plethoric vascular system, rupture is quite probable.

As to treatment, we are quite in the dark, for in this particular case nothing pointed to the spleen as being endangered. Even the renowned Edinburgh Professor could not have detected the slight enlargement that existed, I will venture to say. No local uneasiness was experienced, and nothing save the numbness and bloat of the face before mentioned, suggested the necessity of medication. These two things, together with her robust health, would have indicated to practitioners of the olden times, depletion. Prof. Simpson does not allude to treatment; and he speaks of the enlargement and softening being always connected with an increase of the white corpuscles of the blood. The enlargement was far greater in the cases he recites, than in the one now noticed. In the absence of microscopic examination of the organ, and, in fact, in the absence of a more full and complete account of the condition of the system generally, the treatment in a case fairly made out must be upon general principles. Enlargement of the spleen is common in typhoid fever (laceration sometimes happening); this state of the organ is probably not the disease itself, but only a part of some general condition or derangement of the entire digestive system.—*Boston Med. Journal.*

---

It has been officially decided in Germany that hot baths cause trismus neonatorum.

